# Cytokines, breast cancer stem cells (BCSCs) and chemoresistance

**DOI:** 10.1186/s40169-018-0205-6

**Published:** 2018-09-03

**Authors:** Weilong Chen, Yuanyuan Qin, Suling Liu

**Affiliations:** 1 School of Life Science, The CAS Key Laboratory of Innate Immunity and Chronic Disease, University of Science & Technology of China, Hefei, 230027 Anhui China; 2 Fudan University Shanghai Cancer Center & Institutes of Biomedical Sciences; Cancer Institutes; Key Laboratory of Breast Cancer in Shanghai; Key Laboratory of Medical Epigenetics and Metabolism; Innovation Center for Cell Signaling Network, Shanghai Medical College; Fudan University, Shanghai, 200032 China

**Keywords:** Breast cancer stem cells (BCSCs), Cytokine, Chemoresistance, Tumor microenvironment

## Abstract

Chemotherapy resistance of breast cancer poses a great challenge to the survival of patients. During breast cancer treatment, the development of intrinsic and acquired drug resistance tends to further induce adverse prognosis, such as metastasis. In recent years, the progress of research on cytokine-modulated tumor microenvironment and breast cancer stem cells (BCSCs) has shed light on defining the mechanisms of drug resistance gradually. In this review, we have discussed cytokine regulation on breast cancer chemoresistance. Cytokines can affect tumor cell behavior or reprogram tumor niche through specific signaling pathways, thereby regulating the progress of drug resistance. In addition, we summarized the mutually regulatory networks between cytokines and BCSCs in mediating chemoresistance. Cytokines in the tumor microenvironment can regulate the self-renewal and survival of BCSCs in a variety of ways, sequentially promoting chemotherapeutic resistance. Therefore, the combinational treatment of BCSC targeting and cytokine blockade may have a positive effect on the clinical treatment of breast cancer.

## Introduction

Breast cancer has been seriously endangering the public health because of its high incidence in women [1, 2]. According to the expression of molecular markers (estrogen receptor, progesterone receptor, and HER2), breast cancer can be divided into several subtypes: luminal A, luminal B, HER2+, and triple-negative [3]. According to the different subtypes, there are different therapeutic strategies in clinic. Commonly used treatments for breast cancer include surgery, radiotherapy, chemotherapy, endocrine therapy, targeted therapy and so on. With the development of new drugs, chemotherapy is widely used in the treatment of breast cancer. However, some subtypes of breast cancer are prone to be drug-resistant to chemotherapy, resulting in that the treatment efficacy is very limited, which brings great challenge to clinicians in improving survival of breast cancer patients.

Cancer stem cells are a small population of cells in solid tumors or leukaemia [4, 5]. They are characterized as stem cell-like phenotype, capable of self-renewal and differentiation [6, 7]. Many studies have shown that the role of cancer stem cells can not be ignored in many processes such as tumorigenesis, tumor growth, metastasis and tumor progression [4, 8–13]. In the course of chemotherapy for breast cancer patients, common chemotherapeutic drugs target non-cancer stem cells, but the cancer stem cells can survive and further cause recurrence or even metastasis due to their own characteristics. Therefore, breast cancer stem cells are considered to be the key population leading to drug resistance of breast cancer [14, 15]. With the development of molecular markers of breast cancer stem cells, researchers have found that a small population of cells, selected by markers, can lead to the initiation of tumors, which further confirms the vital role of cancer stem cells in tumor initiation [16, 17].

Cytokine plays important roles in the development of multiple cancers in addition to regulating innate immunity and adaptive immunity, blood cell generation, cell growth and repair of damaged tissue. On one hand, some cytokines can directly regulate the behavior of tumor cells in an either autocrine or paracrine way, and regulate tumor progression, including chemotherapeutic resistance [18, 19]. On the other hand, cytokines can function through affecting other types of cells, such as endothelial cells, fibroblasts and immune cells in the tumor niche. It can further reprogram the tumor microenvironment indirectly, resulting in tumor promotion or tumor suppression and affecting sensitivity to chemotherapeutic agents [20–22]. In addition, studies have shown that some cytokines can also regulate cancer stem cells and further affect the drug resistance of tumor cells [23, 24]. Cancer stem cells can also secrete some specific cytokines and promote their own resistance to chemotherapy drugs to survive [22, 23, 25].

In this review, we summarized recent studies on the roles of cytokines and BCSCs in mediating breast cancer chemoresistance, and discussed the potential of cytokines as therapeutic target to provide new strategies for clinicians to improve the treatment to breast cancer patients.

## Cytokines and chemoresistance

### Direct regulation of cytokines on tumor cells to promote chemoresistance

With the progression of cancer, tumor cells may also express specific cytokine receptors to receive signal stimuli from the corresponding cytokine ligands in the manner of autocrine or paracrine, regulate intracellular signal transduction, and promote the resistance of tumor cells to chemotherapeutic drugs.

#### IL-6

Interleukin-6 (IL-6) was initially identified as B cell stimulating factor 2, enhancing immunoglobulin synthesis through activating B cells and was a prototypical cytokine with pleiotropic and redundant activities of a wide range in immune regulation, hematopoiesis, inflammation and oncogenesis. It helps the host to defend against infection and tissue damage as an inflammatory and immunomodulatory cytokine. However, the persistent IL-6 synthesis leads to the development of various diseases, including cancers [26].

The IL-6 signaling is aberrantly hyper activated in many types of cancer and is generally associated with a poor clinical prognosis [27]. IL-6 was the most highly expressed cytokine in the human colorectal cancer-derived mesenchymal stem cells conditioned medium, and promoted the progression of colorectal cancer cells through IL-6/JAK2/STAT3 signaling, which activated PI3K/AKT signaling. Besides, anti-IL-6 antibody abolished the migration and invasion of colon cancer cells induced by IL-6-activated pathway [28]. Human liver cancer tissues contained high ratio of Tim-3-expressing hepatocytes and HBV involved in Tim-3 upregulation in malignant hepatocytes. The hepatocyte-Tim-3 receptor activates NF-kappa B phosphorylation, which in turn stimulates IL-6 secretion and STAT3 phosphorylation, resulting in tumor growth both in vitro and in vivo [29]. In head and neck cancer, IL-6 can induce its expression as upstream of OPN, and OPN promotes the growth, migration and invasion of cancer cells through activating integrin αvβ3-NF-kappa B axis [30].

**Fig. 1:**
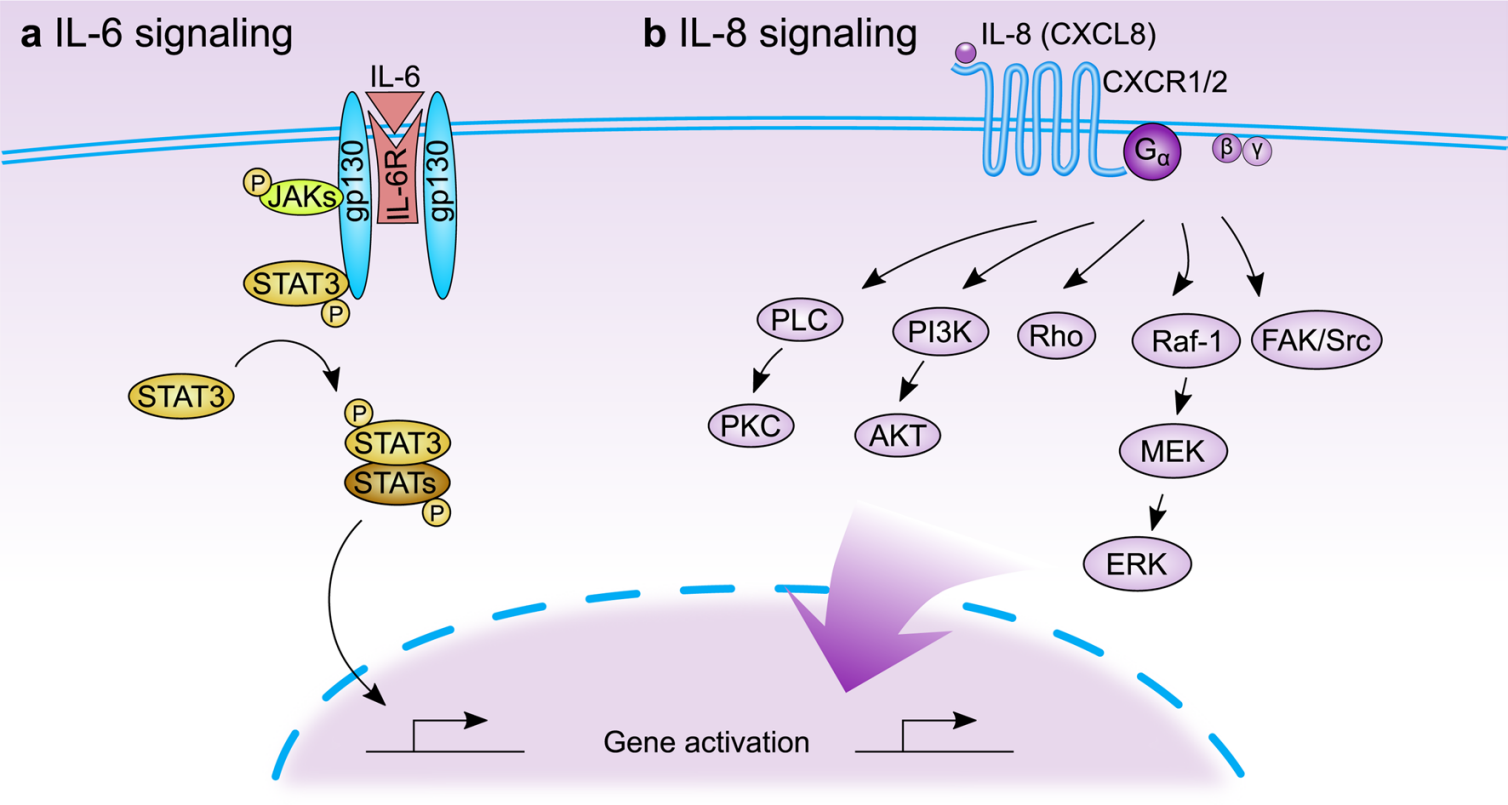
The IL-6 and IL-8 signal transduction pathways. **a** The binding of IL-6 to its receptor IL-6R leads to homodimerization of gp130, resulting in phosphorylation of JAKs which further phosphorylates and activates STAT3. The activated STAT3 binds with other STAT proteins (STATs) to form homodimers or heterodimers, which facilitates the transcription of diverse downstream genes. **b** IL-8 binds to its receptor CXCR1 or CXCR2 (belonging to GPCR superfamily), activating G protein. The G protein subunits activate PLC and PI3K, which further lead to the phosphorylation of PKC and AKT, respectively. Besides, the Rho-GTPase family and non-receptor tyrosine kinases (such as Src and FAK) can be activated by IL-8 signaling. And activated MAPK signaling cascade (Raf-1/MEK/ERK) also transduces the IL-8 stimuli

IL-6 also participated in breast cancer chemoresistance. Local IL-6 paracrine loop act as exogenous IL-6 rich niche for chemo-sensitive breast cancer cells, leading to de novo acquired drug resistance [31]. As one of the principal oncogenic molecules, IL-6 treatment could induce upregulation of HIF-1 alpha via the activation of STAT3, which consequently contributed to its effect against chemotherapeutic drug-induced cytotoxicity and cell apoptosis [32]. In the established MDA-MB-231 metastatic breast cancer cell line, knockdown of endogenous VCAM-1 expression reduced cell proliferation and inhibited IL-6 mediated cell migration, and increased chemosensitivity [33]. Treatment with a genotoxic drug combination (5-fluorouracil, doxorubicin, and cyclophosphamide) activated an NF-kappaB-IL6-dependent inflammatory signaling that imparted stemness to nonstem cancer cells, induced multidrug resistance in breast cancer [34]. The role of JAK-STAT3 in mediating the promotion of IL-6 on drug resistance has been confirmed [26]. IL-6 mainly promotes the expression of multiple genes through STAT3, thus regulating breast cancer drug resistance (Fig. 1a).

#### IL-8

Interleukin-8 (IL-8), also known as chemokine CXCL8, is a cytokine secreted by a wide range of cell populations. IL-8 has cellular chemotaxis to neutrophils to regulate the inflammatory response through combining with chemokine receptor interleukin-8 receptor alpha (IL8RA, also called CXCR1) and interleukin-8 receptor beta (IL8RB/CXCR2), and has multiple cell targets in addition to neutrophils. IL-8 involves in various human diseases, such as atherosclerosis, cancer, inflammatory bowel disease, infection, sepsis, chronic obstructive pulmonary disease, psoriasis and rheumatoid arthritis [35].

The chemokine IL-8 is overexpressed in multiple cancer types, including triple-negative breast cancer (TNBC), where it promotes the acquisition of mesenchymal features, stemness, and chemoresistance. The current research explores the utility of a clinical-stage monoclonal antibody that neutralizes IL-8 as a potential therapeutic option for TNBC [36]. IL-8 showed increased expressions in paclitaxel-treated advanced breast cancer and this over-production effect was inhibited in TLR4-silenced cells. The acquired TLR4-mediated paclitaxel resistance in advanced breast cancer is explained partly by the paracrine effect of IL-8 release [37]. Cytokines produced by breast cancer cells after chemotherapy withdrawal activate both Wnt/beta-catenin and NF-kappa-B pathways, which in turn further promote breast cancer cells to produce and secrete cytokines, forming an autocrine inflammatory forward-feedback loop to facilitate the enrichment of drug-resistant breast cancer cells [38]. Moreover, such an autocrine forward-feedback loop can also be diminished by IL-8 neutralizing antibody or blockade of IL-8 receptors CXCR1/2 with reparixin, and, in a human xenograft model, administration of reparixin after chemotherapy withdrawal effectively attenuates tumor masses [38]. Breast cancer patients treated with chemotherapeutic drugs exhibited poor survival rate and shorter disease-free survival time if their tumor samples expressed high level of IL-8, or its receptor, CXCR1, CXCR2 [38].

The downstream signals activated by IL-8-CXCR1/2 are context-specific, and the effector proteins that mediate IL-8 signals are also pleiotropic, and these effectors may also affect each other. The activation of GPCR (CXCR1 and CXCR2) activated by IL-8 promotes the activation of a variety of downstream pathways, including but may not be limited to PLC-PKC, PI3K-AKT, Rho-GTPase family, FAK/Src of non-receptor tyrosine kinases and MAPK cascade signals (Fig. 1b), which ultimately affect the progression of cancer, angiogenesis, metastasis, and cancer stem cell activation [39]. Although Src and NF-κB have been found to be drug-resistant causes in prostate and colon cancer respectively, the specific mechanism in chemoresistance and critical downstream pathways of IL-8 signal in breast cancer are still not well described, and more research is needed to clarify this point.

#### TGF-β

In addition to the essential roles in germ-layer specification and patterning during embryonic development, transforming growth factor-β (TGF-β) signaling is also involved in human diseases including fibrosis, cardiovascular, cancer, reproductive or wound-healing disorders through control of cellular functions in proliferation, adhesion, invasion, differentiation, apoptosis, and cellular microenvironment [40, 41].

Through analyzing RNA expression in matched pairs of primary breast cancer biopsies before and after chemotherapy, researchers found that biopsies after chemotherapy displayed increased RNA transcripts of TGF-β signaling. And also, in TNBC cell lines and mouse xenografts, the chemotherapeutic drug paclitaxel upregulated autocrine TGF-β signaling [42]. In addition, treatment of TNBC xenografts with LY2157299, the TGF-β type I receptor kinase inhibitor, prevented relapse of tumors after paclitaxel treatment, suggesting that chemotherapy-induced TGF-β signaling enhances tumor recurrence and that TGF-β pathway inhibitors prevent the development of drug-resistant breast cancer cells [42]. Vinorelbine is one of the most active cytotoxic agents in breast cancer, especially metastatic breast cancer and bioinformatics analysis indicated that TGF-β signaling pathway may associate with drug resistance of breast cancer cells to vinorelbine [43].

**Fig. 2:**
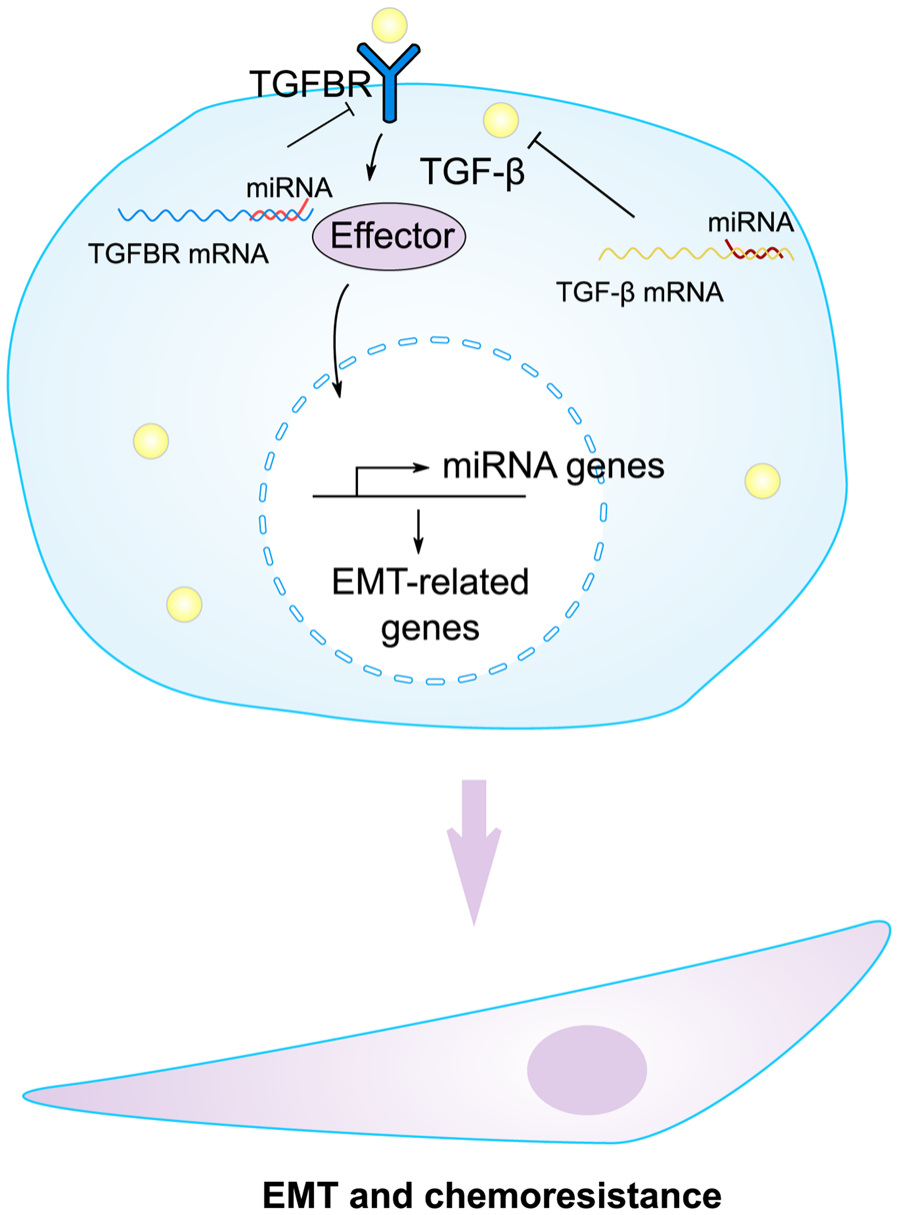
The interplay between microRNAs and TGF-β signaling in regulating chemoresistance of cancer cells. There exists reciprocal modulation between TGF-β and diverse microRNAs. On one hand, TGF-β can affect the transcription of some microRNAs via specific effectors; On the other hand, TGF-β ligand or its receptor TGFBR are the targets of the corresponding microRNAs. These processes control the expression of EMT-related genes, leading to the morphology alteration accompanied with drug resistance in breast cancer

The strong implication of TGF-β in mammary epithelial-to-mesenchymal transition (EMT) promotion is becoming increasingly accepted. PARP3 was upregulated in the course of TGF-β-induced EMT and PARP3 responded to TGF-β-induced signaling to enhance the TG2-Snail-E-cadherin axis during EMT, which demonstrates the critical role of PARP3 in mediating the promotion of TGF-β to EMT [44]. The phenotype switch is now indicated as an important contributor to the acquisition of drug resistance, a clinically relevant issue involved in the preservation of high mortality rates among breast cancer cases [45]. As the regulators of TGF-β, numerous miRNAs are involved in TGF-β signaling [46]. They can intervene in the progression of drug resistance in breast cancer and function as enhancers or inhibitors for chemotherapeutic agents. On the contrary, TGF-β can also regulate the micro-RNA signaling to affect the drug resistance of breast cancer (Fig. 2). TGF-β can induce miR-21 expression, which targeted the 3’ untranslated region of MSH2 mRNA and downregulated its expression. Furthermore, by downregulating MSH2, TGF-β contributed to resistance to DNA-damaging chemotherapy agents (cisplatin, methyl methanesulfonate, and doxorubicin) in breast cancer cells [47].

In addition to IL-6, IL-8 and TGF-β, cytokines which can directly determine the effect of chemotherapy for breast cancer, also includes other factors such as M-CSF, TNFα, IL-1β and so on [18]. They lead to breast cancer resistance to various chemotherapeutic agents including paclitaxel, cisplatin, anthracycline and doxorubicin through differential signaling pathways.

#### Clinical trials in cancer therapies through targeting cytokine signals

These cytokines, closely related to chemotherapeutic resistance, have also attracted the interest of pharmacologists and clinicians, who are trying to develop monoclonal antibodies or small molecular inhibitors targeting these factors to improve the effectiveness of chemotherapies. Therapy through targeting IL-6 signals has been widely applied in inflammatory diseases such as Rheumatoid arthritis [48]. Although no dose-related or cumulative toxicity was apparent in the phase I, open-label study of Siltuximab, an anti-IL-6 monoclonal antibody, in patients of B-cell Non-Hodgkin’s Lymphoma or multiple myeloma (NCT00412321), no responses were seen in patients with relapsed or refractory multiple myeloma treated with single-agent Siltuximab (Table 1). And combining Siltuximab with the bortezomib–melphalan–prednisone (VMP) regimen did not improve complete response (CR), progression free survival, or overall survival but gained very good partial response in multiple myeloma (NCT00911859). In addition, no objective responses were seen in the phase 1/2, multiple-dose, dose-escalation study of siltuximab, suggesting that IL-6 inhibition alone is insufficient in treating advanced or refractory solid tumors including ovarian, pancreatic, colorectal, head & neck cancer and non-small-cell lung carcinoma (NSCLC) (Table 1).

**Table 1:** Clinical trials in cancer therapies related to IL-6, IL-8 and TGF-β signals

Compound and strategy	Cancer type	Phase	Description	Trial numbers	References
Siltuximab; anti-IL-6 mAb	B-cell Non-Hodgkin’s lymphoma, multiple myeloma	Phase I	No dose-related or cumulative toxicity was apparent across all disease indications	NCT00412321	[49]
Siltuximab; anti-IL-6 mAb	Multiple myeloma	Phase II	Randomized study of bortezomib–melphalan–prednisone with or without siltuximab (anti-IL-6) in multiple myeloma	NCT00911859	[50]
Siltuximab; anti-IL-6 mAb	Multiple myeloma	Phase II	The safety and efficacy of siltuximab with or without dexamethasone for patients with relapsed or refractory multiple myeloma	N/A	[51]
Siltuximab; anti-IL-6 mAb	Advanced solid tumors	Phase I/II	Siltuximab monotherapy appears to be well tolerated but without clinical activity in solid tumors	N/A	[52]
Reparixin; CXCR1/2 antagonist	HER-2 negative metastatic breast cancer (MBC)	Phase Ib	Weekly paclitaxel plus reparixin in MBC appeared to be safe and tolerable	NCT02001974	[53]
Reparixin; CXCR1/2 antagonist	Early breast cancer	Phase II	Reparixin 1000 mg t.i.d. for 21 consecutive days appeared to be well tolerated	NCT01861054	[54]
Galunisertib; TGFBR1 inhibitor	Advanced cancer and glioma	Phase I	Based on the safety, PK and antitumor activity in glioma patients, the intermittent administration of LY2157299 at 300 mg/day is safe for future clinical investigation	N/A	[55, 56]
Galunisertib; TGFBR1 inhibitor	Advanced hepatocellular carcinoma (HCC)	Phase II	HCC patients with normal AFP and with TGFβ1 reduction showed improvement in OS compared to patients with non-TGFβ1 reduction	NCT01246986	[57]
Galunisertib; TGFBR1 inhibitor	Recurrent glioblastoma	Phase II	Galunisertib + lomustine failed to demonstrate improved OS relative to placebo + lomustine	NCT01582269	[58, 59]
Galunisertib; TGFBR1 inhibitor	Advanced solid tumors	Phase I	Galunisertib had an acceptable tolerability and safety profile in Japanese patients with advanced cancers	NCT01722825	[60]
Galunisertib; TGFBR1 inhibitor	Pancreatic cancer	Phase II	GG (galunisertib + gemcitabine) resulted in improvement of OS and PFS in patients with PC, with a manageable toxicity profile as compared to GP (gemcitabine + placebo)	NCT01373164	[61]
PF-03446962; Anti-ALK1 mAb	Urothelial cancer	Phase II	They do not recommend further investigation outside of the combination with agents targeting the VEGF receptor axis	NCT01620970	[62]

The data are summarized based on the clinical studies from 2013 to 2018. ALK1 is a member of transforming growth factor-beta (TGF-β) receptor I. Galunisertib, also known as LY2157299 monohydrate

*N/A* not available

CXCR1 is thought to be a receptor selectively expressed in breast cancer stem cells (BCSCs). Reparixin is an allosteric inhibitor of IL-8 (CXCL8) receptor CXCR1/2 has the activity against BCSCs in xenografts of breast cancer [53]. CXCR1 is thought to be a receptor selectively expressed in breast cancer stem cells. Reparixin is an allosteric inhibitor of IL-8 (CXCL8) receptor CXCR1/2 and has the activity against BCSCs in xenografts of breast cancer. It was confirmed that reparixin monotherapy or paclitaxel plus reparixin were appeared to be safe and tolerable in early or metastatic breast cancer (MBC), respectively (NCT01861054, NCT02001974) (Table 1). However, further studies in the clinical trial to observe the action of reparixin on cancer therapy is still needed.

Small molecule inhibitor, LY215799 monohydrate also known as galunisertib, blocks TGF-beta signaling through inhibiting TGFβ receptor I and reduce tumor progression in preclinical models [55]. Besides, Galunisertib has acceptable tolerability and safety in advanced cancer patients (NCT01722825). In advanced hepatocellular carcinoma (HCC), patients treated with Galunisertib showed improvement in overall survival in a phase 2 study (NCT01246986). However, the mono-antibody of ALK1 [a member of transforming growth factor-beta (TGF-β) receptor I], PF-03446962, had no activities as a single drug in refractory urothelial cancer (NCT01620970). The combination of galunisertib and gemcitabine showed improvement of OS and PFS in patients with unresectable pancreatic cancer (PC) compared to gemcitabine + placebo (NCT01373164). Unfortunately, in patients with recurrent glioblastoma, Galunisertib plus lomustine failed to demonstrate improved OS relative to placebo plus lomustine (NCT01582269) (Table 1). In general, it is still very promising to improve the therapeutic effect of cancers via blockade of TGF-β signaling, which requires more clinical studies to confirm.

### Indirect influence of cytokines on tumor chemoresistance via remodeling tumor microenvironment

The tumor microenvironment (TME) comprises immune system elements (such as macrophages and lymphocytes), fibroblast, cells composing blood vessels, myofibroblast, mesenchymal stem cells, adipocytes and extracellular matrix (ECM). Tumor microenvironment (or the tumor niche) plays a vital role in the progression of cancer [63–68], and affects many processes such as tumor growth, metastasis, relapse and drug resistance [69–73].

#### Cytokines and macrophages

Tumor-associated macrophages (TAM) are the prominent components of TME in breast cancers. Macrophages exhibit a high plasticity in response to various external signals and participate in innate and adoptive immune responses to control numerous factors of TME [74]. Depending on the microenvironmental signal present, macrophages undergo different types of activation, including the “classic” pro-inflammatory phenotype (also called M1) and the “alternative” anti-inflammatory phenotype (also called M2) or even in the transitional state between these two kinds of macrophages. TAMs closely resemble the M2-polarized. Clinicopathological studies have suggested that TAM accumulation in tumors correlates with a poor clinical outcome [74]. However, the characteristics of tumor-infiltrated macrophages are complex. TAMs show pleiotropic effects on tumor behavior due to be stimulated by differential cytokines. Some chemokines may increase the infiltration of TAM and form suitable conditions for tumor outgrowth. Once infiltrated, macrophages may also be regulated by cytokines, changing the gene expression, releasing factors that are beneficial to the progression of tumor and the factors associated with immunosuppression. Finally, multiple behaviors of macrophages affected by cytokines can remodel the tumor microenvironment and promote breast cancer chemotherapy resistance.

Breast cancer-associated macrophages express high levels of insulin-like growth factors 1 and 2 (IGFs) and are the main source of IGFs within both primary and metastatic tumors [73]. In total, 75% of breast cancer patients show activation of insulin/IGF-1 receptor signaling and this correlates with increased macrophage infiltration and advanced tumor stage. In addition, blockade of IGF in combination with paclitaxel showed a significant increase in chemosensitivity of tumor compared to paclitaxel monotherapy [73]. TAMs and its supernatants significantly prevent breast tumor cells from apoptosis caused by paclitaxel and the high level of IL-10 secreted by TAMs was responsible for drug resistance of breast cancer [75]. The possible TAMs-modulated drug resistance mechanism involved may be associated with elevation of bcl-2 gene expression and up-regulation of STAT3 signaling in tumor cells, forming IL-10/STAT3/bcl-2 signaling axis accounting for chemoresistance of breast cancer [75].

#### Cytokines and fibroblasts

The presence of cancer-associated fibroblasts (CAFs) was found in almost all solid tumors. However, their abundance varies widely among different types of cancer. For example, breast, prostate and pancreatic cancer contain more CAFs, while CAFs in brain, kidney and ovarian tumors is rare. CAFs are markedly different from tumorigenic malignant cells [76]. They have undergone epithelial-to-mesenchymal transition and show fibroblast-like morphology. In addition, there is a great difference between cancer cells and CAF cells that the karyotype is relatively stable, and there are few genetic alterations in CAFs. They are defined as all the fibroblastic, nonneoplastic, nonvascular, nonepithelial, and noninflammatory cells found in a tumor [77].

CAFs may lead to the resistance of cancer cells to anti-tumor drugs by means of soluble factors. Chemokine- or cytokine-signaling pathway perhaps drive CAF to remodel the extracellular matrix (ECM) in the tumor microenvironment and the CAF-remodeled ECM can provide favorable soil for the change of EMT characteristics of tumor cells, conferring chemotherapeutic resistance [78, 79]. The EMT process usually promotes chemoresistance by inducing cell cycle arrest or altering the expression of transporters enabling chemotherapeutic drugs uptake of tumor cells [80–82]. Stimuli from proinflammatory cytokine IL-6 expressed by tumor cells, is sufficient to induce the expression of Twist1 in normal fibroblasts and transdifferentiate them into CAFs through the activation of STAT3 pathway [83]. In the xenograft tumor model of breast cancer, ectopic expression of IL-6 can significantly increase the infiltration of Twist1-positive CAFs, and Twist1 is necessary and sufficient for the trans-differentiation formation of CAFs. In addition, CXCL12 is the target gene of Twist1 transcriptional regulation as the downstream signaling [83]. These studies elucidate that cytokine (IL-6) can promote the remodeling of tumor microenvironment by promoting CAFs formation, infiltration and promoting downstream cytokines expression.

The collagen type I secreted by CAFs was found to reduce the uptake of chemotherapeutic drugs in the tumor, and therefore plays a vital role in regulating the chemosensitivity of cancer to multiple forms of chemotherapy [84]. Accordingly, targeting CAFs can increase the drug absorption inside the tumor, inhibit growth and metastasis of primary tumor cells, and the growth of breast cancer that are resistant to multiple drugs in mice [84]. Taken together, cytokines affect the formation and infiltration of CAFs, and CAFs can reshape the characteristics of ECM in microenvironment and subsequently influence the drug resistance of breast cancer cells. On the other hand, CAFs can secrete specific cytokines to further reprogram the microenvironment.

### Synergic regulation of multiple cytokines on chemoresistance

Due to the complexity of the tumor microenvironment, the diversity of cytokines and the existence of pleiotropic regulatory networks among them, the effects of cytokines on tumor progression are often not independent on each other [85–87]. Similarly, in the regulation of cytokines on chemotherapy resistance in breast cancer, some cytokines will exhibit synergistic or inter-dependent phenomena. It is possible that specific context-dependent cytokines can simultaneously regulate the same or different signaling pathways in breast cancer to promote drug resistance; or a cytokine from breast cancer cells or stromal cells in microenvironment promotes the production of another cytokine from the tumor cells; it may also be the combination of the two cytokines above and the more complex cascade response.

Previous studies have shown that cisplatin treatment can significantly alter the secretory phenotype and behavior of mesenchymal stromal cells (MSCs). After cisplatin treatment, the MSCs not only changed the phosphorylation level of many kinases, but also increased the secretion of IL-6 and IL-8, which increased the chemotherapeutic resistance of breast cancer cells to cisplatin [88]. TGF-β can promote paclitaxel resistance in triple-negative breast cancer which is mediated by SMAD4 [42]. In addition, SMAD4 regulates taxane resistance by promoting the expression of IL-8 [42]. The formation of TGF-β-SMAD4-IL-8 axis in breast cancer cells shows a synergistic regulation of multiple cytokines in chemotherapeutic resistance of breast cancer. In brief, in promoting breast cancer chemotherapy resistance, sometimes cytokines are not “fighting” alone. In response to the various effects (necrosis, apoptosis and senescence) on tumor cells produced by chemotherapeutic stimulation, the tumor cells and other cells in TME secrete pleiotropic cytokines, which act on themselves or other cells in manner of autocrine or paracrine, and jointly promote the survival of tumor cells under the treatment of anti-tumor drugs.

## Interplay between cytokines and BCSCs on chemoresistance

### BCSCs drive chemoresistance

BCSCs are a special group of cells in breast cancer, which can maintain self-renewal and differentiation. The therapeutic effect of treatments to advanced breast cancer, such as radiotherapy and chemotherapy, is prone to be limited because of the presence of BCSCs in the tumor, and they may further lead to recurrence, metastasis and chemoresistance [89–94].

In 2003, CD24^−^CD44^+^lineage^−^ cells were identified as BCSCs for the first time, which had a strong ability to initiate breast tumor, which was confirmed and used as a biomarker for BCSCs repeatedly [95]. Aldehyde dehydrogenase (ALDH) is a family of enzymes performing the function of the oxidation of intracellular aldehydes to carboxylic acids and retinoic acid (RA). Ginestier et al. found that in normal and malignant mammary cells, about 8% of normal mammary epithelial cells showed the activity of ALDH identified by ALDEFLUOR assay [16]. In addition, breast cancer cells with the ALDH activity (ALDH^+^) identified by ALDEFLUOR assay can initiate the xenograft tumor with as little as 500 cells. Even 50,000 ALDH-negative breast cancer cells from the same tumor can not initiate the xenograft tumor. Moreover, if the ALDH^+^CD24^−^CD44^+^ phenotype, as little as 20 breast tumor cells can form breast tumor [16]. Besides, many evidences from other solid tumors also indicate that ALDH can serve as the marker of CSCs [89, 96–100]. Therefore, CD24^−^CD44^+^ and ALDH^+^ are the most commonly used biomarkers for identification and isolation of BCSCs.

**Fig. 3:**
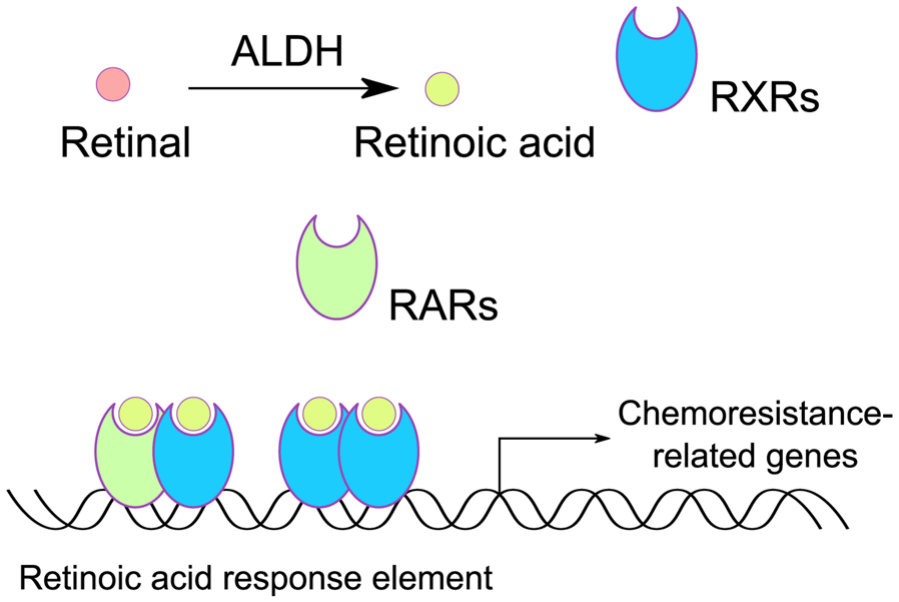
Retinoic acid signaling catalyzed by ALDH modulates the chemoresistance of cancer cells. Retinal is oxidized into retinoic acid by ALDH and then binds with RARs or RXRs. Activated RAR and RXR forms heterodimer to bind with retinoic acid response element, promoting the transcription of retinoic acid-responsive genes of which some play a vital role in driving chemotherapeutic resistance. In addition, RXRs can assemble into a homodimer to modulate the transcription while RARs cannot

RA produced from aldehyde oxidation catalyzed by ALDH can bind to the nuclear retinoic acid receptors (RARs) or retinoid X receptors (RXRs). The heterodimer formed by RARs and RXRs recognizes a direct repeat of a 6 base pair DNA sequence motif with a 2 or 5 base pair spacer at the promoter region of many retinoid-inducible genes [101, 102]. In addition, RXR also can bind to DNA and regulate gene expression and cell differentiation as a homodimer [101]. ALDH can regulate gene expression through RAR signaling pathway, explaining the reason why ALDH plays an important role in the survival and differentiation of BCSCs (Fig. 3). Furthermore, the reprogramming of ALDH on genes expression profiling related to drug effects (genes involved in ABC transporter, DNA damage repair, clearance of reactive oxygen species) also explains the effect of ALDH^+^ BCSC on chemotherapeutic resistance [103].

A lot of signaling pathways in cells increasing stemness property, cell re-population, and self-renewal of BCSCs, indirectly promote BCSC-mediated chemoresistance of breast cancer [90, 93, 94, 104–109]. These studies provide evidences that in promoting breast cancer chemoresistance, BCSCs resemble a “hub” integrating drug resistance signalling. It can directly defend against the cytotoxic effects of anticancer drugs, or it can also be triggered by other signals to drive breast cancer cell resistance.

### Cytokines promote chemotherapy resistance through enhancing BCSCs

Several studies have shown that IL-6 can regulate the self-renewal of BCSCs [110]. Furthermore, a stable equilibrium between BCSC and non-stem breast cancer cells was maintained by the amount of IL-6 secreted by BCSCs, expression level of IL-6 receptor and the whole response of non-BCSC to IL-6 [110]. Mechanistically, IL-6-JAK1-STAT3 signaling axis plays a key role in the conversion from non-BCSCs to BCSCs through regulating the expression of OCT4 gene [111]. In the study of Ibrahim et al, Syndecan-1 (CD138), a heparan sulfate proteoglycan, promotes the breast cancer stem cell phenotype via regulation of IL-6/STAT3 signaling pathways [112]. And Lin et al showed that tanshinone IIA has the potential of targeting and killing BCSCs, and can inhibit BCSCs growth in vivo and in vitro by downregulating the IL-6/STAT3/NF-κB signaling pathway [113]. These studies suggest that the promotion of IL-6 to BCSCs is generally achieved through activation of STAT3, but the effector signaling associated with the BCSCs property caused by the STAT3 activity are variegated because of the differences in the research object and the experimental system.

In addition, BCSCs rely on activated Notch signals to maintain cell survival and proliferation, and the activation of this pathway is closely related to the growth of breast tumors [114]. Inhibition of Notch1 in tumors can significantly inhibit tumor outgrowth, which is caused by promoting apoptosis and reducing the frequency of BCSCs [115]. In addition to Notch signaling, other embryonic signals are also important for stem cell regulation, such as Hedgehog and Wnt [116–119]. These pathways are closely related to the regulation of IL-6 and their interactions with IL-6 also affect the characteristics of breast cancer stem cells. In conclusion, given the critical role of BCSCs in conferring chemoresistance, enhancement of IL-6 to BCSCs population reveals the clew of that it can promote the resistance of breast cancer cells to chemotherapeutic drugs [34].

In addition to IL-6, some studies suggested that IL-8, CCL2 and TGF-β can also promote the characteristics of BCSCs and give rise to chemotherapeutic resistance [38, 42, 120–123].

## Therapeutic implications of interfering the interactions between cytokines and BCSCs in chemoresistance

**Fig. 4:**
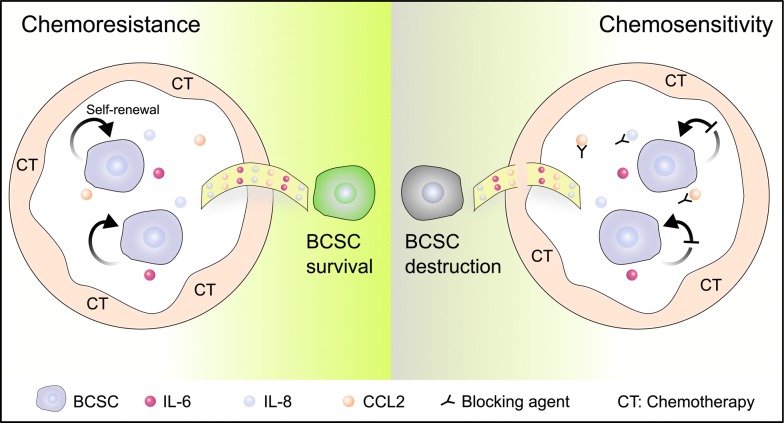
The interplay of cytokines and BCSCs on regulating chemotherapeutic resistance. Under the stress of chemotherapeutic drugs, a variety of different sources of cytokines can regulate the self-renewal and promote the survival of BCSCs, leading to drug resistance. Cytokines are a convenient “bridge” between BCSCs and chemoresistance (left panel). If the regulatory signaling to BCSCs is interfered by cytokine blockade, the support of cytokine signals for BCSC chemo-resistance will be broken, increasing the sensitivity of breast cancer chemotherapy (right panel)

The interaction between cytokines and BCSCs is pivotal for the formation of chemotherapy resistance. Cytokines can act on the surface receptors on breast cancer stem cell membrane through autocrine or paracrine, and promote the proliferation, survival and self-renewal of BCSCs. In response, BCSCs may also increase the secretion of specific factors under the stress of chemotherapeutic drugs to regulate their survival signaling. The augment in the levels of specific cytokines will trigger the response of non-tumor cells in tumor microenvironment, such as secreting cytokines to remodel the BCSCs niche, which may be more conducive to the survival of BCSCs (Fig. 4). When BCSCs are influenced by cytokines, the expression of genes affiliated with resistance may be altered, such as the increase in the level of transporter protein, expression of anti-apoptotic genes, and activation of DNA repair genes. Eventually, BCSCs and specific cytokines collude in doing evil as chemotherapy resistance through reciprocal modulation.

Therefore, interfering the interaction between BCSC and cytokines may bring great benefits to the improvement of chemotherapy resistance in breast cancer. In view of the role of IL-8 in mediating BCSC self-renewal and breast cancer chemoresistance, several IL-8 releasing inhibitors, small-molecule CXCR1/2 inhibitors and neutralizing antibodies against IL-8 and CXCR1/2 have been reported during the past two decades [68]. Combination of blocking IGF signaling and paclitaxel can significantly reduce tumor cell proliferation and pulmonary metastasis compared with treatment of paclitaxel alone [73]. This provides a theoretical basis for the further development of combinatorial strategy of paclitaxel and IGF blockers in the treatment of invasive breast cancer. Moreover, IGF-positive stromal cells may be designated as potential biomarker for auxiliary diagnosis [73].

## Conclusion and perspective

Chemotherapy resistance in breast cancer is often accompanied with alterations of specific cytokine levels in tumor tissues. The mutual regulation between these cytokines and BCSCs has weakened the effect of chemotherapy and reduced the survival of breast cancer patients.

There also exists great heterogeneity in the populations of BCSCs, which leads to limited therapy efficacy of targeting BCSCs directly [124]. Taking into account the necessity of cytokines for BCSC self-renewal and survival, and the combinational therapeutic strategy of targeting BCSCs and neutralizing cytokines may bring great hope to the improvement of the survival for breast cancer patients (Fig. 4).

## Abbreviations

TME: tumor microenvironment
BCSCs: breast cancer stem cells
JAK2: Janus kinase 2
STAT3: signal transducer and activator of transcription 3
PI3K: phosphatidylinositol-4,5-bisphosphate 3-kinase
AKT: AKT serine/threonine kinase
Tim-3: Hepatitis A virus cellular receptor 2
HBV: Hepatitis B virus
NF-kappa B: nuclear factor kappa B
OPN: secreted phosphoprotein 1
HIF-1: hypoxia inducible factor 1
VCAM-1: vascular cell adhesion molecule 1
TLR4: toll like receptor 4
CXCR: C-X-C motif chemokine receptor
GPCR: G protein-coupled receptors
TGF-ß: transforming growth factor beta
EMT: epithelial-to-mesenchymal transition
MSH2: MutS homolog 2
M-CSF: colony stimulating factor 1
TNFα: tumor necrosis factor
ECM: extracellular matrix
TAM: tumor-associated macrophages
IGFs: insulin-like growth factors
CAF: cancer-associated fibroblasts
Twist1: twist family bHLH transcription factor 1
CXCL12: C-X-C motif chemokine ligand 12
MSCs: mesenchymal stromal cells
SMAD4: SMAD family member 4
ALDH: aldehyde dehydrogenase
RA: retinoic acid
RARs: retinoic acid receptors
RXRs: retinoid X receptors
ABC: ATP binding cassette
